# A genetically-engineered von Willebrand disease type 2B mouse model displays defects in hemostasis and inflammation

**DOI:** 10.1038/srep26306

**Published:** 2016-05-23

**Authors:** Frédéric Adam, Caterina Casari, Nicolas Prévost, Alexandre Kauskot, Cécile Loubière, Paulette Legendre, Christelle Repérant, Dominique Baruch, Jean-Philippe Rosa, Marijke Bryckaert, Philip G. de Groot, Olivier D. Christophe, Peter J. Lenting, Cécile V. Denis

**Affiliations:** 1 Institut National de la Santé et de la Recherche Médicale, UMR_S 1176, Univ. Paris-Sud, Université Paris-Saclay, 94276 Le Kremlin-Bicêtre, France; 2 Kyoto Gakuen University, Kameoka, Japan; 3 INSERM UMR_S1140, 75006 Paris, France; 4 University Paris-Descartes, 75006 Paris, France; 5 Department of Clinical Chemistry and Haematology, University of Utrecht, University Medical Center Utrecht, Utrecht, The Netherlands

## Abstract

von Willebrand disease (VWD)-type 2B is characterized by gain-of-function mutations in the von Willebrand factor (VWF) A1-domain, leading to increased affinity for its platelet-receptor, glycoprotein Ibα. We engineered the first knock-in (KI) murine model for VWD-type 2B by introducing the p.V1316M mutation in murine VWF. Homozygous KI-mice replicated human VWD-type 2B with macrothrombocytopenia (platelet counts reduced by 55%, platelet volume increased by 44%), circulating platelet-aggregates and a severe bleeding tendency. Also, vessel occlusion was deficient in the FeCl3-induced thrombosis model. Platelet aggregation induced by thrombin or collagen was defective for KI-mice at all doses. KI-mice manifested a loss of high molecular weight multimers and increased multimer degradation. In a model of VWF-string formation, the number of platelets/string and string-lifetime were surprisingly enhanced in KI-mice, suggesting that proteolysis of VWF/p.V1316M is differentially regulated in the circulation *versus* the endothelial surface. Furthermore, we observed increased leukocyte recruitment during an inflammatory response induced by the reverse passive Arthus reaction. This points to an active role of VWF/p.V1316M in the exfiltration of leukocytes under inflammatory conditions. In conclusion, our genetically-engineered VWD-type 2B mice represent an original model to study the consequences of spontaneous VWF-platelet interactions and the physiopathology of this human disease.

Von Willebrand disease (VWD) is a congenital bleeding disorder originating from quantitative or qualitative defects in von Willebrand factor (VWF), a multimeric glycoprotein essential for primary hemostasis^1^. Indeed, following vascular injury, platelet adhesion to the subendothelium and platelet thrombus formation is critically dependent on VWF, which forms a molecular bridge between subendothelial components such as collagens and platelet receptors, the glycoprotein GPIb-IX-V receptor complex and integrin αIIbβ3^2^. VWD can be classified into 3 major types^3^. VWD types 1 and 3 are defined by partial and near complete quantitative deficiencies in VWF, respectively. The various VWD type 2 subtypes are due to qualitative defects of the VWF protein. The most intriguing VWD subtype is VWD-type 2B characterized by gain-of-function mutations in the VWF A1 domain, which comprises the binding site for GPIbα^4^. In contrast to wild-type (WT) VWF, VWF-type 2B variants interact spontaneously with platelets *via* GPIbα causing patients to experience, somewhat counter-intuitively, hemorrhage but not thrombosis^5^. This bleeding phenotype is probably due to both a loss of high molecular weight VWF multimers (the most functionally effective VWF forms) and, secondly, variable thrombocytopenia^5^. The predominant hypothesis is that high molecular weight VWF multimers are adsorbed onto platelets and that both components are simultaneously cleared from the bloodstream, thus explaining their concomitant plasma decrease in VWD-type 2B patients^6^. Previously, our group and the group of Dr. D. Lillicrap group reported a novel VWD-type 2B murine model based on the hydrodynamic injection of murine VWF cDNA bearing type 2B mutations into VWF-deficient mice^7^,^8^. This mouse model has been instrumental in furthering our understanding of the pathological mechanisms underlying VWD-type 2B. We were able to demonstrate that the decrease in circulating high molecular weight VWF multimers was due to increased proteolysis of mutant VWF by ADAMTS13^8^, the protease responsible for regulating VWF multimer size. In addition, increased platelet clearance was also found to be a likely contributor to thrombocytopenia in these mice^9^. Finally, using these mice as well as platelets from patients, we were able to demonstrate that the type 2B VWD p.V1316M mutation also caused defective platelet αIIbβ3 integrin activation, demonstrating that the bleeding tendency of VWD-type 2B patients is likely to be multifactorial^10^.

The use of hydrodynamic injection to generate transient models of VWD-type 2B has been extremely informative and has the advantage of making the study of phenotypic inter-variability between various type 2B VWD mutations possible. However, such approach has also numerous drawbacks such as ectopic VWF synthesis (hepatic instead of endothelial and megakaryocytic origin), transient VWF expression and multimeric pattern with a predominance of low and intermediate forms^11^,^12^. To circumvent these limitations, we decided to knock the p.V1316M mutation into the murine *Vwf* locus in order to create a *bona fide* genetic VWD-type 2B mouse model. These mice display human VWD-type 2B-like characteristics, which make them a promising model to investigate the pathological consequences of abnormal VWF-GPIbα interactions, observed not only in the context of VWD-type 2B but also in other pathologies where spontaneous VWF-platelet interactions occur.

## Results

### Vwf/p.V1316M mice are viable and fertile

**Figure 1:**
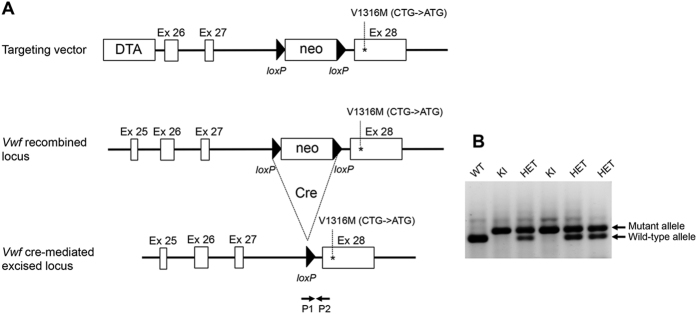
Engineering of the Vwf/p.V1316M mice. (**A**) Targeting vector comprising a genomic fragment of *Vwf* with exons 26, 27 and mutated exon 28. A neomycin positive selection cassette flanked by two *loxP* sites was inserted upstream of exon 28 and a Diphteria toxin negative selection cassette was inserted outside the homologous recombination area, upstream of exon 26. Following homologous recombination and double selection of embryonic stem cells, the recombined locus now bears the mutated exon 28 preceded by a neomycin flanked by two *loxP* sites. After injection of ES cells into blastocysts and breeding of the generated mice with C57BL/6 Cre delete mice, the neomycin cassette was excised and heterozygous mice carrying the mutant allele were generated. (**B**) Genotyping results of intercrossing of heterozygous mice carrying one mutant allele. Position of the genotyping primers (P1 and P2) is indicated in panel (A). The PCR products are 380 bp in length for the wild-type allele and 485 bp for the mutant allele.

By using the targeting strategy outlined in Fig. 1, the p.V1316M mutation was introduced in the mouse genome. Starting from mice heterozygous for the p.V1316M mutation, three colonies were generated (wild-type (WT-), heterozygous (HET-) and homozygous (KI-) mice) in order to allow direct comparison between mice of different genotype. Intercross of HET-mice yielded the expected Mendelian distribution of *Vwf*/*p.V1316M* genotypes (not shown), demonstrating that the p.V1316M mutation does not impair murine embryonic development or perinatal survival.

### VWD-type 2B KI-mice exhibit normal VWF antigen levels, macrothrombocytopenia and abnormal multimer pattern

VWF antigen levels were similar between all three genotypes and did not change significantly during the 14‒16 months of follow-up (Supporting information Fig. S1).

**Figure 2:**
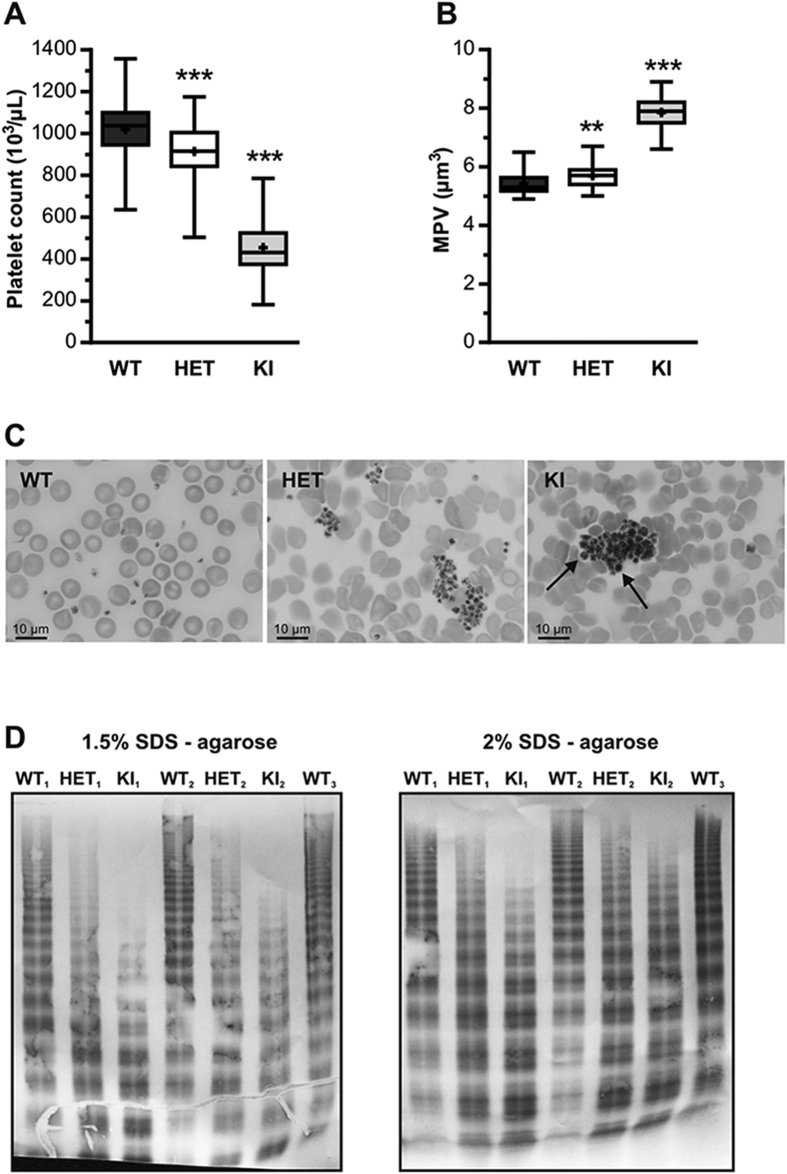
Platelet counts, blood smear analysis and VWF multimeric patterns of WT-, HET- and KI-mice. (**A**) Platelet count and (**B**) mean platelet volume (MPV) were determined in WT (n = 58), HET (n = 73) and KI (n = 52) mice. Data are presented in a box-and-whiskers plot and statistical significance was evaluated by 1-way ANOVA followed by Dunnett’s test (**p < 0.01; ***p < 0.001). (**C**) Representative blood smears of WT, HET and KI mice after May-Grünwald Giemsa staining. (**D**) Multimer composition of circulating VWF proteins in WT-, HET- and KI-mice. Gels represent analysis of plasma samples using 1.5%- and 2% SDS-agarose electrophoresis.

We next determined platelet counts in mice of each colony. Platelet count for HET- (914 ± 133 × 10^3^/μL; n = 73) was modestly but significantly reduced compared to WT-mice (1019 ± 154 × 10^3^/μL; n =  = 58; *p* < 0.0001; 1-way ANOVA followed by Dunnett’s test; Fig. 2A). In contrast in KI-mice, platelet count was markedly decreased (455 ± 139 × 10^3^/μL; n = 52; *p* < 0.0001; 1-way ANOVA followed by Dunnett’s test; Fig. 2A). A long-term follow up for up to 16 months revealed that the difference in platelet counts between WT-, HET- and KI-mice remained relatively steady over time. Of note, an increase in platelet counts was detected in WT-mice over time (Supporting information Fig. S2).

Platelet volumes were slightly increased for HET- (5.7 ± 0.3 μm^3^) and more markedly for KI-mice (7.8 ± 0.6 μm^3^) compared to WT-mice (5.4 ± 03 μm^3^) (*p* = 0.001 and *p* < 0.0001 for HET- and KI-mice, respectively; 1-way ANOVA followed by Dunnett’s test; Fig. 2B). Similar to platelet counts, no major difference in platelet volume was detected in a long-term follow-up study (Supporting information Fig. S3).

The presence of larger platelets could be visualized in blood smears of KI-mice (arrows, Fig. 2C). Moreover, blood smears revealed the presence of platelet aggregates for HET- and KI-mice but not WT-mice. We also analyzed multimer patterns for each of the colonies. For WT-mice, a full multimer range was observed similar to that of regular C57BL/6 mice, whereas HET- and KI-mice present with increased low- and intermediate-molecular weight multimers (MWMs) and reduced high MWM numbers, although this reduction was more pronounced for KI- than HET-mice when visualized via 1.5% SDS-agarose electrophoresis (Fig. 2D). Moreover, analysis via 2% SDS-agarose electrophoresis revealed an increase in the quantity of satellite bands for HET- and KI-mice, suggesting increased degradation by ADAMTS13 (Fig. 2D). Thus, KI-mice display a VWD-type 2B-like phenotype with reduced platelet counts, increased platelet volume, reduced high-MWMs and excessive VWF degradation. A similar phenotype, although less pronounced, was apparent for HET-mice.

### Defective hemostasis and thrombosis in Vwf/p.V1316M mice *in vivo*

**Figure 3:**
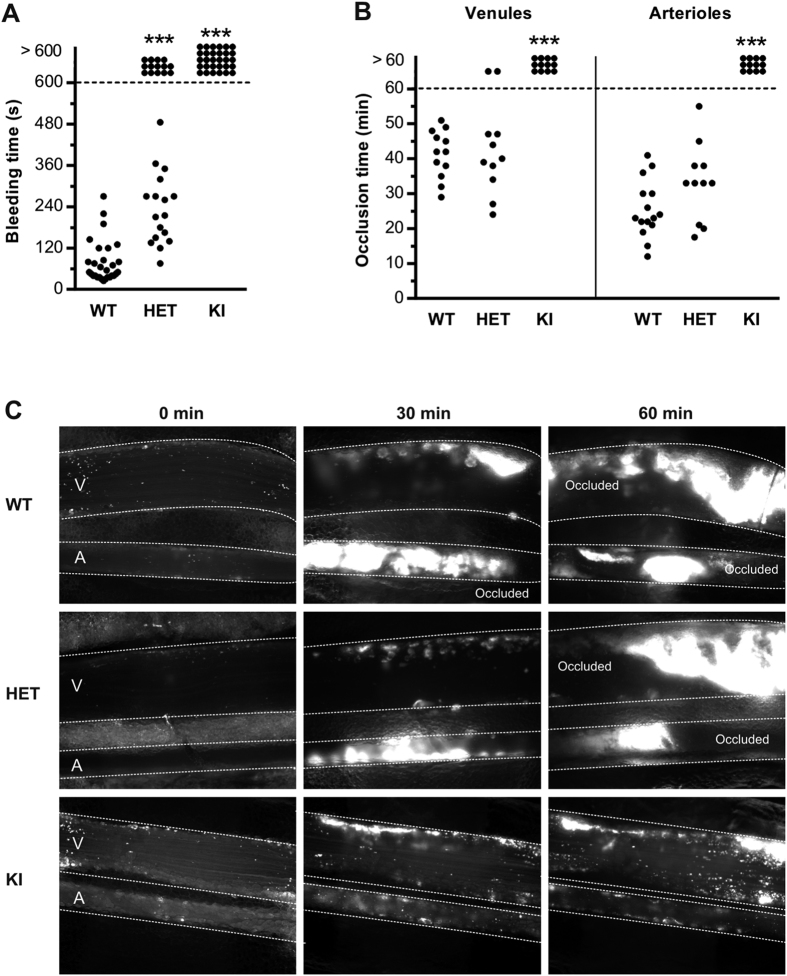
HET- and KI-mice display abnormal *in vivo* hemostasis. (**A**) Tail bleeding time in WT-, HET- and KI-mice. Each dot represents one individual. (**B**) *In vivo* thrombosis model in mesenteric vessels (venules and arterioles) of WT-, HET- and KI-mice after FeCl_3_-induced injury. Adhesion and thrombus formation of fluorescently-labeled platelets were monitored by intravital videomicroscopy. Graph represents the occlusion time of the vessels. Statistical significance was determined by 2-tailed Mann-Whitney test (***p < 0.001). (**C**) Representative images of the thrombotic process in mice of the three different genotypes.

The hemostatic activity of *Vwf*/*p.V1316M* mice was assessed in two distinct models. First, in a tail clip-bleeding assay, a severe bleeding phenotype was observed for KI-mice: none of the mice (n = 30) stopped bleeding within the 10-min observation period, whereas an average bleeding time of 78 ± 11 s (n = 29; *p* < 0.0001; 2-tailed Mann-Whitney test) was detected for WT-mice (Fig. 3A). For HET-mice, a heterogeneous bleeding phenotype was observed: 14 out of 31 mice tested did not stop bleeding, whereas bleeding was contained for the remaining 17 mice (*p* < 0.0001 *vs* WT; 2-tailed Mann-Whitney test). It should be noted that the 17 HET-mice that did stop bleeding, had a prolonged bleeding time (234 ± 25 s) compared to WT-mice (Fig. 3A). We also subjected these animals to FeCl_3_-induced vessel injury, monitoring thrombus formation in mesenteric venules and arterioles arteries. HET-mice (n = 11) were similar to WT-mice (n = 15) in terms of occlusion time in venules and arterioles (Fig. 3B,C). In contrast, no occlusion of venules or arterioles within the 60-min observation period was detected for KI-mice (n = 12; Fig. 3B,C). Animals homozygous for the p.V1316M mutation experience severe hemostatic and thrombotic defects *in vivo*. In contrast, only hemostasis was affected in heterozygous animals while the thrombosis response was normal.

### The presence of the p.V1316M mutation impairs platelet adhesion *in vitro*

**Figure 4:**
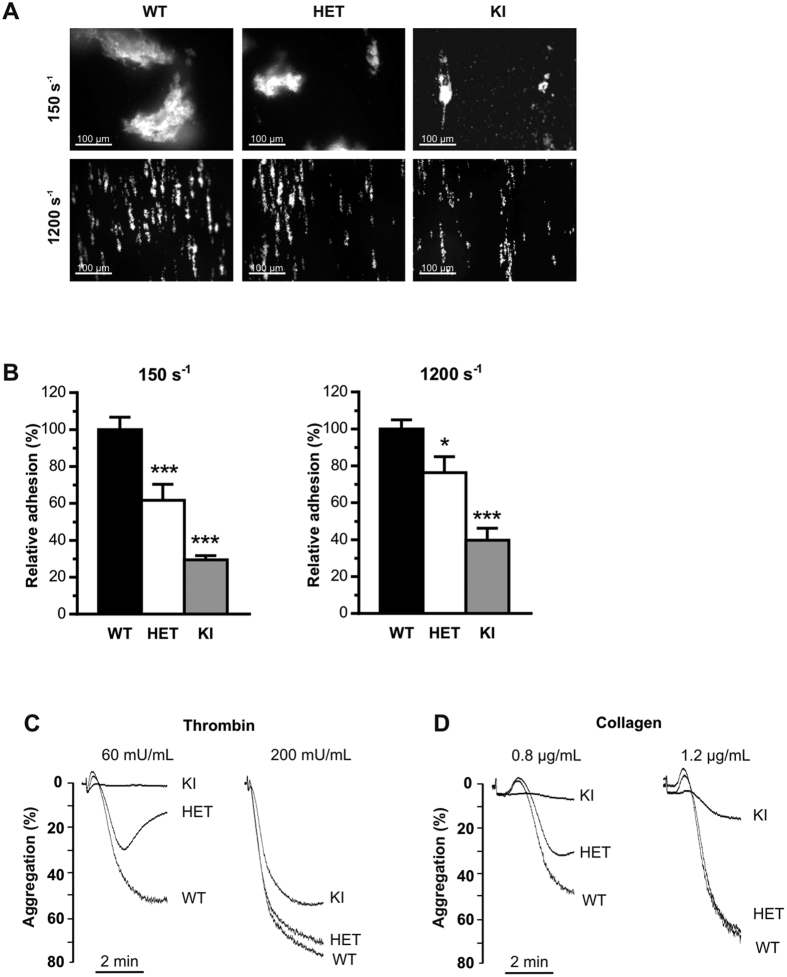
HET- and KI-mice display decreased *in vitro* platelet adhesion. *In vitro* platelet adhesion was measured in a whole-blood perfusion assay for WT-, HET- and KI-mice over a fibrillar collagen matrix at venous (150 seconds^−1^) or arterial (1200 seconds^−1^) shear rates. (**A**) Representative images of platelet adhesion under blood flow. (**B**) Platelet adhesion was quantitated by assessment of the mean percentage of the total area covered by platelets and the results are expressed as relative adhesion to WT, given as 100% ± S.E.M of at least 3 independent experiments. Statistical significance was determined by 1-way ANOVA followed by Dunnett’s test (*p < 0.05; ***p < 0.001). Scale bar represents 100 μm. (**C**,**D**) Aggregation of washed (3 × 10^8^/mL) WT-, HET- and KI-mouse platelets induced by thrombin (60 or 200 mU/mL; (**C**)) or collagen (0.8 or 1.2 μg/mL; (**D**)). Traces are representative of at least 3 independent experiments. Results are expressed as the percent change in light transmission with respect to the blank (buffer without platelets), set at 100%.

To investigate the capacity of VWF/p.V1316M to support platelet adhesion, anticoagulated blood from WT-, HET- and KI-mice was perfused over a collagen surface under venous and arterial shear conditions (150 s^−1^ & 1200 s^−1^, respectively). Platelet counts were not normalized prior to perfusion. For KI-mice, platelet adhesion was markedly reduced under both conditions (29.4 ± 2.3%; *p* < 0.0001; 1-way ANOVA followed by Dunnett’s test) and 39.7 ± 6.5%; *p* < 0.0001; 1-way ANOVA followed by Dunnett’s test) residual adhesion compared to WT respectively, Fig. 4A,B). A significant reduction in platelet adhesion was also found for HET-mice under both venous and arterial shear conditions: 61.8 ± 8.5% (*p* < 0.0004) and 76.3 ± 8.7% (*p* = 0.0354; 1-way ANOVA followed by Dunnett’s test) residual adhesion compared to WT, respectively (Fig. 4A,B). Measure of fluorescence intensity, reflecting thrombus size, was also similarly decreased in HET- and KI-mice compared to WT-mice. This defective platelet adhesion strongly suggests that platelet function is affected, especially when considering the results obtained with HET-blood where platelet count is near-normal.

### Platelet function is impaired in Vwf/p.V1316M mice

Our perfusion assay results, together with our recent study showing that exposure of platelets to VWF/p.V1316M resulted in impaired platelet aggregation due to defective integrin αIIbβ3 activation^10^ prompted us to investigate platelet function in *Vwf*/*p.V1316M* mice. Reduced platelet aggregation was observed for HET-mice compared to WT-mice at low thrombin and collagen concentrations (50% reduction with thrombin and 36% reduction with collagen; Fig. 4C). However, reduced platelet aggregation for HET-mice was normalized at higher agonist concentrations. Platelets from KI-mice were irresponsive to either thrombin or collagen at low agonist concentrations, and displayed impaired aggregation at high agonist concentration (30% reduction with thrombin and 82% reduction with collagen). These data confirm that the p.V1316M mutation is associated with reduced platelet responses, a defect that can only further exacerbate impaired hemostasis in HET- and KI-mice.

### Platelet adhesion to VWF strings is increased in Vwf/p.V1316M mice

**Figure 5:**
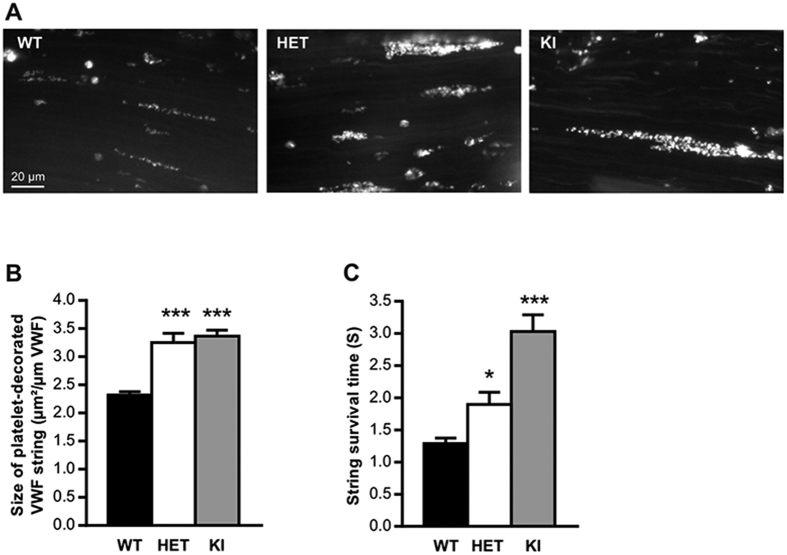
*In vivo* VWF/platelet string formation. *In vivo* platelet-decorated VWF strings on the surface of mesenteric endothelium of WT-, HET- or KI-mice after topically application of FeCl_3_ solution (10%). (**A**) Images represent “platelet strings” attached to an injured vein. (**B**) The size of platelet-decorated VWF strings was calculated by dividing the length of each “platelet string” by their surface. (**C**) The lifetime of platelet-decorated VWF strings was determined per mouse and it was designated as “string survival time” (SST). In total, at least 200 platelet-decorated VWF strings were analyzed for each genotype. Statistical significance was determined by 1-way ANOVA followed by Dunnett’s test (*p < 0.05; ***p < 0.001). Scale bar represents 20 μm.

Stimulated release of VWF from endothelial cells is associated with the formation of platelet-decorated VWF strings, which are rapidly cleaved by ADAMTS13. Since VWD-type 2B mutations promote increased susceptibility to ADAMTS13-mediated cleavage^13^, it was of interest to investigate VWF/platelet string formation in mice expressing Vwf/p.V1316M. Application of a FeCl_3_ solution to mesenteric venules stimulates the formation of such strings^14^. VWF/platelet strings could be observed in WT-, HET- and KI-mice. Interestingly, quantitative analysis of images revealed that the number of platelets *per* string was significantly increased by 40% and 45% for HET- and KI-mice respectively compared to WT-mice (*p* < 0.0001; 1-way ANOVA followed by Dunnett’s test; Fig. 5). Similarly *in vitro*, platelet adhesion to VWF strings released from endothelial cells was also strongly increased when endothelial cells from a VWD-type 2B patient were used (Supporting information Fig. S4).

Surprisingly, the string lifetime was prolonged for both HET-mice (by 47%; *p* = 0.040; 1-way ANOVA followed by Dunnett’s test) and KI-mice (by 134%; *p* < 0.0001; 1-way ANOVA followed by Dunnett’s test) compared to WT-mice. To check whether different levels of ADAMTS13 activity could explain this result, we measured ADAMTS13 activity in mice of the three genotypes. No difference was found between WT-, HET- and KI-mice (Supporting information Table S1).

### Leukocyte recruitment is increased in Vwf/p.V1316M mice in the reverse passive Arthus reaction

**Figure 6:**
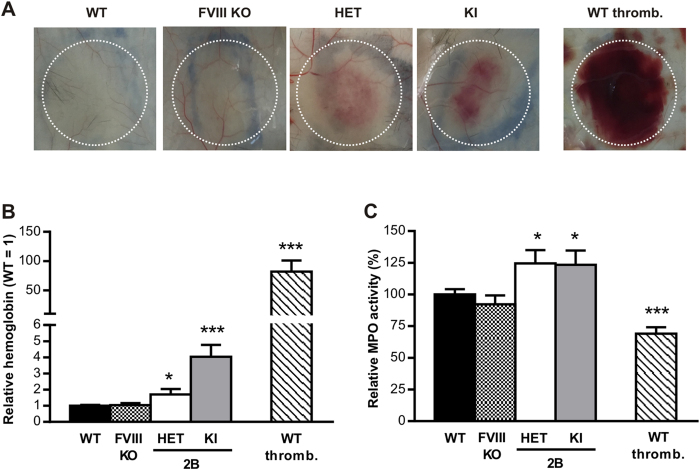
Reverse passive Arthus reaction. Comparison of hemoglobin content (**A**,**B**) and leukocyte infiltration (**C**) in biopsies of the inflamed skin of WT-, HET-, KI-mice, FVIII-deficient mice (FVIII KO) and platelet-depleted WT-mice (WT thromb.) after 4 hours of the reverse passive Arthus reaction. In (**A**), representative photographs are shown. The results are expressed as relative hemoglobin to WT ± S.E.M (**B**) and as relative MPO activity to WT, given as 100% ± S.E.M (**C**) of 5–11 independent experiments. Statistical significance was determined by 1-way ANOVA followed by Dunnett’s test (*p < 0.05, ***p < 0.001).

Based on recent data reporting a role for VWF-GPIbα interaction in leukocyte extravasation^15^, we also subjected the VWD-type 2B mice in an *in vivo* inflammation model. We used the reverse passive Arthus reaction since this model is bridging together inflammation and hemostasis. As previously described^16^, no hemorrhage could be detected in inflamed skin of WT-mice. Haemorrhage was also absent in mice deficient for coagulation factor VIII (Fig. 6). In HET- and KI-mice however, a respective increase in hemoglobin content of 1.7-fold (*p* = 0.0205; 1-way ANOVA followed by Dunnett’s test) and 4-fold (*p* < 0.0001; 1-way ANOVA followed by Dunnett’s test) was measured, revealing blood leakage in the surrounding tissues (Fig. 6A,B). A profound hemorrhage (100-fold more compared to WT-mice) was detected in WT-mice with severe thrombocytopenia (<5% residual platelets; Fig. 6A,B). The measure of myeloperoxydase activity in inflamed skin biopsies was similar for WT-mice and haemophilic mice and significantly reduced by 40% (*p* < 0.0001; 1-way ANOVA followed by Dunnett’s test) in the platelet-depleted WT-mice (Fig. 6C). Interestingly, myeloperoxydase activity was significantly higher in HET- and KI-mice (23% and 25%, respectively; *p* = 0.048 and *p* = 0.036; 1-way ANOVA followed by Dunnett’s test), reflecting a phenotype-specific increase in leukocyte recruitment at injury site in these mice (Fig. 6C).

## Discussion

The VWF-GPIbα axis has long been recognized as critical to initial platelet adhesion during vascular injury under conditions of high shear rate, thereby participating in primary hemostasis^17^. This has been exemplified by a number of hemorrhagic disorders where this interaction was either absent (VWD-type 3, Bernard-Soulier syndrome), decreased (VWD-type 2A or 2M) or even increased (VWD-type 2B, platelet-type VWD)^1^,^3^,^18^,^19^. A number of regulatory mechanisms are involved in the modulation of the VWF-GPIbα interaction^20^. For one, VWF needs to adopt an “active” conformation or platelet-binding conformation for binding to GPIbα to occur^21^. In the context of a vascular injury, this conformational change occurs when VWF binds to exposed sub-endothelial vascular collagens^22^. However there are a number of pathological situations where inappropriate VWF-GPIbα interactions can occur. This involves diseases in which circulating VWF is in an “active” conformation such as thrombocytopenic thrombotic purpura, malaria or HELLP syndrome^23^. Genetic mutations may also result in abnormal processing of the VWF protein, leading to constitutive platelet binding, as seen in VWD-type 2B for example^5^. In the last few years, several studies have reported adverse biological consequences stemming from altered VWF-GPIbα interactions such as impaired megakaryopoiesis^24^, platelet apoptosis^25^ or osteopetrosis^26^. Most observations have been originating from studies on VWD-type 2B, the prototypical disease model for the presence of such active VWF. To facilitate *in vivo* analysis, we and others have previously employed the hydrodynamic injection (HI)-based VWD-type 2B model^7^,^8^. However, to bypass limitations of the HI model, we generated a congenital murine model of VWD-type 2B and chose to express the p.V1316M mutation which accounts for 10% of all VWD-type 2B patients in the French cohort of VWD (CRMW: Centre de Référence de la Maladie de Willebrand, coordinators: Profs Agnès Veyradier and Jenny Goudemand). Patients bearing this specific mutation are difficult to treat, often requiring concomitant infusion of VWF concentrates and platelets. Our reasoning was that selecting a mutation capable of eliciting such a severe form of VWD-type 2B would be more informative on the long-term consequences of spontaneous VWF-GPIbα interactions *in vivo*.

Our genetically engineered mice display a distinct VWD-type 2B phenotype, severe for the homozygous KI-mice and more moderate for the HET-mice. They recapitulate characteristic symptoms of the human disease, such as formation of large platelets, presence of platelet aggregates in blood smears and lack of high-MWM of VWF^5^. Interestingly, we were able to detect a number of differences in this model compared to the HI-based model. Indeed, in the genetically engineered mice, we find that: (a) circulating plasma levels of VWF are in the normal range (*vs* 508–970% in HI-model) and stay constant over time; (b) thrombocytopenia is less pronounced (455 × 10^3 ^platelets/μL *vs* <300 × 10^3 ^platelets/μL in HI-model); (c) the increase in platelet volume is more pronounced despite higher platelet count (7.8 μm^3^ *vs* 7.1 μm^3^ in HI-model); (d) high-MWM are absent in all mice tested (*vs* 50% of mice in HI-model); (e) VWF is present in storage pools (endothelial cells, platelets) or in the sub-endothelial compartment (but not in the HI model); (f) the presence of Weibel-Palade bodies allows formation of platelet-decorated VWF strings (an event that does not occur in the HI-model).

Of interest, the notion that the full picture of VWD-type 2B is present only in homozygous KI-mice suggests that this mutation is less severe in the murine context compared to the human situation, where the heterozygous state is sufficient to induce a profound thrombocytopenia. Human VWF A1 domain poorly interacts with murine GpIb, suggesting that the three dimensional structures of the murine and human A1 domains are not fully identical. This dissimilar structure could cause the p.V1316M mutation being less severe in the murine structure compared to the human structure. Another explanation could relate to the VWF-GpIb ratio in mice and humans. Human platelets contain approximate 25,000 copies of GpIb/platelet at a platelet count of 150–400 × 10^3 ^platelets/μL, corresponding to 4–10 million copies of GpIb/μL^27^. Murine platelets contain 36,000 copies of GpIb/platelet at a platelet count of 800–1000 × 10^3 ^platelets/μL, corresponding to 28–37 million copies of GpIb/μL^28^. From our own unpublished observations, we know that VWF:Ag is similar between humans and C57BL/6 mice. Therefore, the ratio VWF/GpIb is reduced >3-fold in mice compared to humans. This could explain why higher concentrations of mutated VWF subunits (*i.e.* homozygous *versus* heterozygous) are needed to induce a severe VWD-type 2B phenotype.

Another result that was unexpected was that VWF antigen levels were similar for control and VWF/p.V1316-expressing mice. Human VWD-type 2B patients are characterized by a wide range of VWF-antigen levels (<30–200%) that overlap the normal range (50–200%)^29^, indicating that normal antigen levels do not exclude VWD-type 2B. This is also true for human VWF/p.V1316M, the antigen levels of which vary between 27% and 65% of normal in patients belonging to the French CRMW-cohort. The milder effect of the murine p.V1316M variant compared to the human mutation could account for the antigen levels remaining unaffected.

When subjected to *in vivo* FeCl3-induced thrombosis, KI-mice showed no discernable vein or arteriole occlusion while HET-mice were found to occlude normally (Fig. 3B,C). In contrast, both KI- and HET-mice displayed a more or less severe bleeding phenotype in the tail clip-bleeding model, despite HET-mice having a normal platelet count (Fig. 3A). Similarly, *in vitro* whole blood perfusion assay showed that platelet adhesion to collagen was significantly reduced for both HET- and KI-mice and this was observed at both venous and arterial shear rates (Fig. 4A,B). Since platelet counts had not been normalized in this experiment in order to mirror *in vivo* conditions, the decrease in platelet adhesion observed in KI-mice may be due, in part, to lower circulating platelet numbers (Fig. 2A). However, the fact that HET-platelets displayed decreased adhesion (Fig. 4A,B) despite normal platelet counts suggests that a qualitative platelet defect is also involved. Indeed, both HET- and KI-platelets had abnormal responses to collagen and thrombin when tested in an aggregometer (Fig. 4C,D). This thrombocytopathy no doubt participates to the observed hemorrhagic complications. The platelet dysfunction observed in this mouse model is in agreement with our previous report on the thrombocytopathy associated with VWD-type 2B^10^. Furthermore, these data are compatible with the phenotype described for the Montreal Platelet Syndrome, in which patients display a platelet defect, while carrying the same p.V1316M mutation^30^.

Another question that we were interested in answering was whether our KI-mice would display increased VWF proteolysis by ADAMTS13, as previously reported for VWF-type 2B mutants^13^. We performed multimeric analysis of plasma VWF isolated from WT-, HET- and KI-mice and were able to detect a marked increase in proteolytic VWF fragments in both HET-and KI-mice (Fig. 2D). This confirms that, at least in plasma, VWF-type 2B is more susceptible to ADAMTS13-cleavage. In plasma, proteolysis may occur while VWF is being exposed to elongational flows and/or increased laminar shear rates^21^,^31^ and the importance of this pathway is illustrated by the rapid appearance (within 15 min) of VWF degradation products following the infusion of recombinant non-proteolyzed VWF in VWD-type 3 patients^32^. Our multimer result does not address the question of whether this increased susceptibility also applies to VWF assembled into platelet-binding strings following secretion from stimulated endothelial cells. We therefore tested our animals using the VWF/platelet string assay originally described by André *et al*.^33^ and modified by De Meyer *et al*.^14^. We first observed that VWF strings released from human or murine VWD-type 2B endothelial cells were extremely reactive as significantly higher number of platelets were adhering *per* string compared to WT-derived strings. In some examples, we were able to visualize several layers of platelets agglutinated to each other either in VWD-type 2B endothelial cells (Fig. S4) or in HET- and KI-mice (Fig. 5A). *In vivo*, such strings have a short survival time due to rapid cleavage of freshly-released VWF by ADAMTS13^33^. To our surprise, both HET- and KI-strings had a longer survival time than WT-strings, reflecting decreased cleavage by ADAMTS13, in apparent contrast with the increased cleavage of circulating VWF-type 2B. At this point, we can only speculate as to the mechanism(s) that reduce the efficiency of ADAMTS13-mediated proteolysis of these strings: a) the high number of platelets present on the VWF strings may impede ADAMTS13 accessibility to the VWF cleavage site; b) VWF self-association may be stimulated by the presence of VWD-type 2B mutations as it was previously reported for shear conditions^34^ and thicker VWF strands could be more resistant to ADAMTS13 cleavage. *In vitro* analysis by us and Scaglione *et al*.^35^ revealed that VWF-type 2B variants are indeed prone to more efficient self-association compared to wt-VWF (Supplementary Fig. S5) or c) the truncated ADAMTS13-variant that is expressed in C57Bl/6J mice^36^ could react less efficiently with VWF/p.V1316M compared to WT-VWF.

Besides its established role in hemostasis, there is mounting evidence linking VWF to inflammation and leukocyte recruitment^37^,^38^,^39^. The formation of VWF-platelet complexes and VWF-GPIbα interactions more specifically appear to play an important role in leukocyte extravasation^15^. We therefore proceeded to measure leukocyte recruitment in our Vwf/p.V1316M mice using the reverse passive Arthus reaction, a local inflammation model based on the presence of immune complexes. We observed that not only hemorrhage was increased in HET- and KI-mice but also leukocyte recruitment was significantly increased (Fig. 6). We considered the possibility that the increased leukocyte recruitment was a result from passive leakage during hemorrhage. However, leukocyte recruitment was reduced in platelet-depleted mice (<5% residual platelets), which displayed severe hemorrhage in this model (Fig. 6). A similarly reduced leukocyte recruitment in the presence of exaggerated blood loss has previously reported for GpVI-deficient mice, arguing against a passive transfer of leukocytes under hemorrhagic conditions^40^. Together, our data strongly point to an active role of the VWD-type 2B mutant in the recruitment of leukocytes in this model. The mechanism by which VWF-type 2B contributes to this pro-inflammatory phenotype remains to be determined, but two hypotheses could be of relevance in this regard. First, we have previously shown that botrocetin-activated VWF binds directly *via* its A1 domain to PSGL-1^38^. It is possible that the presence of the VWF/p.V1316M mutation that turns on the active conformation of VWF promotes binding to PSGL-1, thereby favoring leukocyte-rolling and subsequent extravasation. Second, as mentioned above, Petri and colleagues have reported that the formation of VWF/platelet complexes is essential for optimal leukocyte extravasation^15^. It seems conceivable that the preformed VWF/platelet complexes in the context of VWD-type 2B provide an environment that allows more efficient or perhaps exaggerated leukocyte extravasation compared to conditions where WT-VWF is present.

In conclusion, we have developed the first congenital murine model of VWD-type 2B. We have now validated this model by showing that it recapitulates human VWD-type 2B in terms of thrombocytopenia as well as defective hemostasis and thrombosis. An interesting point is that in humans, VWD-type 2B is of dominant inheritance and is usually present in a heterozygous state. In the murine model, although HET-mice do display some features of the disease, only homozygous-KI-mice totally phenocopy the human clinical picture. Despite these human-mouse differences, our model has contributed to identify new avenues that will be explored further in the future such as impaired megakaryocytopoiesis and the pro-inflammatory phenotype. In addition, this murine model will also be useful to test new treatment options for VWD-type 2B, such as the use of agents blocking VWF-GPIbα, aptamers^41^ or others.

## Methods

### Reagents

Fibrillar collagen (equine type I) was obtained from Kordia (Leiden, The Netherlands). Apyrase (grade VII), bovine serum albumin (BSA), hexadecyltrimethylammonium bromide, rhodamine 6G, bovine thrombin and ferric chloride were obtained from Sigma (Saint-Quentin-Fallavier, France). d-Phe-Pro-Arg chloromethylketone dihydrochloride (PPACK) was purchased from Calbiochem-VWR (Fontenay-sous-Bois, France). Polyclonal anti-human VWF antibody was from Dako (Les Ulis, France). Immunoglobulins (IgG) anti-BSA were from MP Biomedicals (Illkirch, France). Mixture of purified rat monoclonal antibodies directed against mouse GPIbα were from Emfret Analytics (Eibelstadt, Germany). Peroxidase-labeled anti-human Fc or anti-HPC4 antibodies were from Sanquin (Amsterdam, the Netherlands) and Roche (Meylan, France), respectively.

### Animal statement

Housing and experiments were done in accordance with French regulations and the experimental guidelines of the European Community. This project was approved by the local ethical committee of Université Paris-Sud (comité d’éthique en experimentation animale no. 26) under the number 2012-039.

### Engineering of the VWD-type 2B knock-in mouse model

The generation of the VWD-type 2B knock-in mouse model was entirely outsourced to genOway (Lyon, France). *Vwf* genomic fragments were retrieved by PCR from a bacterial artificial clone library of C57BL/6 mouse strain (Welcome Trust Sanger Institute).

A targeting vector was constructed from a genomic fragment containing exons 26, 27 and 28 of *Vwf.* A single base pair modification was inserted in exon 28 in order to reproduce a VWD-type 2B mutation (p.V1316M) (Fig. 1A). A neomycin positive selection cassette flanked by two *loxP* sites was inserted upstream of exon 28 and a Diphteria toxin negative selection cassette was inserted outside the homologous recombination area, upstream of exon 26. The final targeting vector was linearized with PmeI and introduced in C57BL/6-derived embryonic stem (ES) cells by electroporation. Individual ES clones were screened for homologous recombination by both Southern blot and PCR. The presence of the desired mutation was assessed by sequencing of the PCR product. Targeted clones (4 out of 230 clones isolated) were injected into blastocysts derived from an albino C57BL/6 mouse strain. Chimeric males thus generated were bred with C57BL/6 Cre deleter mice to excise the *loxP* flanked neomycin cassette and give rise to heterozygous mice carrying the mutant allele. Breeding of heterozygous mice yielded both wild-type and homozygous mutant VWD-type 2B offspring. Genotying was performed through ear biopsy and the Kapa mouse genotyping kit (Kapa Biosystems, Wilmington, MA) processing with PCR primers P1 (TGTGGCTAGAGACATAGATTGGAAGGAAATC) and P2 (CGTAAGGTTCACACCATCACAGTGACTGTAG) (Fig. 1A) leading to a 380 bp PCR product size for wild-type allele and 485 bp PCR product size for mutant allele (Fig. 1B).

### Determination of endogenous murine ADAMTS13 activity and VWF antigen levels

For ADAMTS13 activity, blood was collected in lithium-heparin tubes. After preparation of plasma, samples were diluted 6.7-, 10-, and 20-fold in 50 μL 0.005% Tween-20, 25 mM CaCl_2_, 5 mM BisTris (pH 6.0), and 50 μL of FRETS-VWF73 substrate (diluted 1:25 in the same buffer; Peptanova GmbH) was added. Fluorescence (excitation at 340 nm, emission at 440 nm) was monitored for 60 minutes at a 5-minute interval at 30 °C. For VWF antigen, blood was collected on EDTA (10 mM final concentration) via retro-orbital puncture using heparinized glass capillaries, which avoids activation. Platelet poor plasma was then prepared by centrifuging blood samples for 20 minutes at 1500*g*. Plasma samples were kept at −80 °C until use. Normal mouse plasma (NMP) and samples from individual mice were prepared using the same method.

### Long-term follow-up platelet counts and volume and VWF antigen levels

Eight to ten mice of each three genotypes (WT, HET and KI) were selected for monitoring over the course of a year. Blood sampling was performed under isoflurane anesthesia every two months *via* venous puncture. Blood counts were determined with an automatic cell counter (Scil Vet ABC Plus, Horiba Medical, France). Mice were kept for 14–16 months.

### VWF multimeric structure analysis

The multimeric structure of VWF was analyzed from mouse plasma by 0.1% SDS and 1.5% or 2% agarose (Seakem^r^ HGT Agarose; Lonza Walkersville) gel electrophoresis as described^42^. Multimers were visualized using an alkaline phosphatase-conjugated anti-human VWF polyclonal antibody.

### Mouse platelet preparation

Mice were anesthetized by intraperitoneal injection of sodium pentobarbital (60 mg/kg), then whole blood was collected by cardiac puncture into 80 μM PPACK and 10% ACD buffer (124 mM sodium citrate, 130 mM citric acid, 110 mM dextrose, pH 6.5). Isolated platelets were obtained as previously described^43^.

### Platelet aggregation

Platelet aggregation was monitored by measuring light transmission through the stirred suspension of washed WT, HET or KI platelets (3 × 10^8^/mL) at 37 °C using a Chronolog aggregometer (Chrono-log Corporation, USA)^43^. Platelet aggregation was triggered by adding thrombin or collagen at indicated doses. Representative traces for aggregation were obtained from at least three independent experiments. Results are expressed as the percent change in light transmission with respect to the blank (buffer without platelets), set at 100%.

### *In vitro* thrombus formation under flow conditions

Thrombus formation was evaluated in a whole-blood perfusion assay on a collagen matrix (50 μg/mL) under different shear conditions, as previously described^43^.

### Measure of bleeding time

Bleeding time assays were performed on 8- to 12-week old mice by cutting off the tip of the tail (3 mm from the tip) and immediately immersing it in saline. We then recorded the time taken for the bleeding to stop. Tail bleeding was monitored for at least 60 seconds beyond this time point, to ensure that bleeding did not start again. Tail bleeding assays were stopped at 600 seconds if the bleeding did not stop^43^.

### Platelet aggregation

Platelet aggregation was performed using washed platelets obtained from WT-, HET-, and KI-mice (all normalized at 3 × 10^8^/mL) as detailed in the supplementary information. Platelet aggregation was triggered by adding thrombin or collagen at indicated doses.

### Ferric chloride-induced thrombosis model

Ferric chloride (FeCl_3_) injury was induced in 4- to 5-week-old mice, as previously described^44^ with slight modifications. To facilitate visualization of thrombus formation, platelets of anesthetized mice were fluorescently labeled *in vivo* by injection of rhodamine 6G (3.3 mg/kg) into the retro-orbital plexus. After topical deposition on the mesenteric vessels of FeCl_3_ solution (10%), thrombus growth was monitored in real-time with an inverted epifluorescent microscope (×10) (Nikon Eclipse TE2000-U).

### *In vivo* platelet adhesion to secreted VWF by endothelial cells

*In vivo* platelet-decorated VWF strings were evaluated as previously described with slight modifications^14^. Briefly, mice were anesthetized by intraperitoneal injection of sodium pentobarbital (60 mg/kg), then platelets were fluorescently labeled *in vivo* by injection of rhodamine 6G (3.3 mg/kg) into the retro-orbital plexus. After exposure of the mesenteric blood vessels, a filter paper saturated with FeCl_3_ solution (10%) was applied topically on a mesenteric microvessel. Vessels were scanned to localize a region with visible platelet-decorated VWF strings. These strings were then recorded in real-time during the following 6 minutes with an inverted epifluorescent microscope (x20) (Nikon Eclipse TE2000-U) coupled to Metamorph 7 software (Universal Imaging Corporation). During that time frame, no thrombus formation occurred. For each experiment, the lifetime of at least 20 platelet-decorated VWF strings was determined per mouse and designated “string survival time” (SST). Moreover, the size of platelet-decorated VWF strings was calculated by dividing the length of each “platelet string” by their surface. In total, at least 200 platelet-decorated VWF strings were analyzed for each genotype.

### Reverse passive Arthus reaction

Shaved mice were anesthetized by inhalation of isoflurane and the reverse passive Arthus reaction (rpA) was initiated by intravenous injection of BSA (75 μg BSA/g mouse) immediately followed by intradermal injection of IgG anti-BSA (60 μg)^16^. Four hours after injection, skins were harvested using a biopsy punch (8 mm) and tissues were assessed for hemorrhage and leukocyte recruitment. In addition to the VWD-type 2B mice, also factor VIII-deficient mice were used^45^. When indicated, thrombocytopenia was induced 5 minutes before induction of the rpA reaction. Such platelet depletion was achieved in mice by intravenous injection of a mixture of purified rat monoclonal antibodies directed against mouse GPIbα at 1.5 μg/g body weight, leading to more than 95% reduction in circulating platelets at 4 hours post-injection.

### Hemoglobin tissue analysis

Skin biopsies were blended in 50 mM potassium phosphate buffer and centrifuged (10 min, 2000 *g*, 4 °C). Hemoglobin content of the supernatants was measured by adding formic acid and absorbance reading at 405 nm.

### Determination of myeloperoxidase (MPO) activity

MPO activity was measured as previously described^46^. Briefly, skin biopsies were blended in 50 mM potassium phosphate buffer supplemented with 50 mM hexadecyltrimethylammonium bromide, sonicated and centrifuged (10 min, 13000 *g*, RT). After centrifugation, MPO activity was assessed in the supernatant by adding tetramethylbenzidine and absorbance reading at 450 nm after stopping the reaction with sulfuric acid (0.5 N).

### Statistical analysis

Statistical significance was evaluated with Student’s *t* tests, two-tailed Mann-Whitney U-tests or 1-way ANOVA followed by Dunnett’s test as indicated, using GraphPad Prism (San Diego, CA).

## Additional Information

**How to cite this article**: Adam, F. *et al*. A genetically-engineered von Willebrand disease type 2B mouse model displays defects in hemostasis and inflammation. *Sci. Rep.* **6**, 26306; doi: 10.1038/srep26306 (2016).

## Supplementary Material

Supplementary Information
