# Supplementary material for: A genetically-engineered von Willebrand disease type 2B mouse model displays defects in hemostasis and inflammation
Source: Sci Rep. 2016 May 23;6:26306. doi: 10.1038/srep26306 (PMC4876317; doi:10.1038/srep26306)
Supplement: Supplementary Information [file srep26306-s1.pdf]

SREP-16-08580 Supplementary information

A genetically-engineered von Willebrand disease type 2B mouse model displays defects in hemostasis and inflammation

Frédéric Adam[1], Caterina Casari[1], Nicolas Prévost[2], Alexandre Kauskot[1], Cécile Loubière[1], Paulette Legendre[1], Christelle Repérant[1], Dominique Baruch[3,4], Jean-Philippe Rosa[1], Marijke Bryckaert[1], Philip G. de Groot[5], Olivier D. Christophe[1,*], Peter J. Lenting[1], Cécile V. Denis[1]

[1]Institut National de la Santé et de la Recherche Médicale, UMR_S 1176, Univ. Paris-Sud, Université Paris-Saclay, 94276 Le Kremlin-Bicêtre, France
[2]Kyoto Gakuen University, Kameoka, Japan
[3]INSERM UMR_S1140, 75006 Paris, France
[4]University Paris-Descartes, 75006 Paris, France
[5]Department of Clinical Chemistry and Haematology, University of Utrecht, University Medical Center Utrecht, Utrecht, The Netherlands.

Supplementary Results:

VWF antigen levels over time :

There was no difference in VWF antigen levels between WT-, HET- and KI-mice. Levels averaged from 61% to 103% compared to a C57BL/6 murine plasma set at 100%.

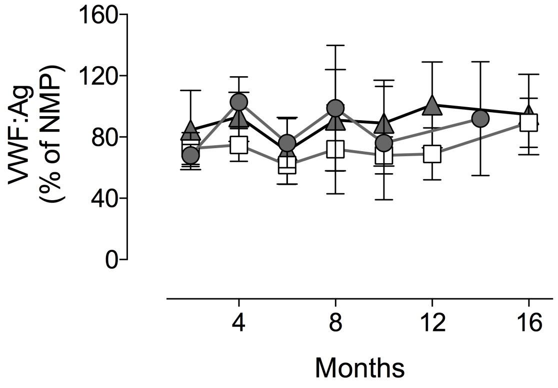

Figure S1 : Follow-up of VWF:Ag over time. Means ± SD measured in WT (triangles), HET (open squares) and KI-(circles) mice. NMP: Normal mouse plasma.

Platelet counts over time :

When looking at how platelet counts were evolving over time, we did not see any statistical differences in HET and KI (p=0.15 and p=0.39 ; 1-way ANOVA followed by Dunnett's test) mice between 2 and 14-16 months. In WT mice, a trend analysis performed *via* ANOVA revealed that platelet counts were increasing over time (p=0.0005).

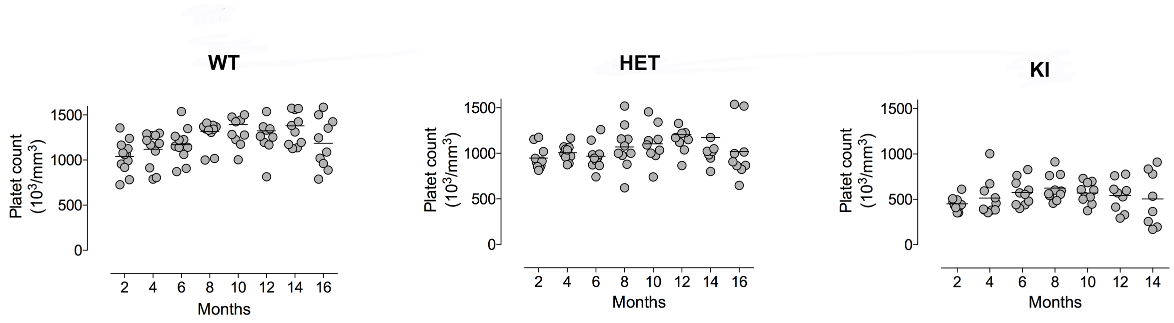

Figure S2 : Follow-up of platelet counts over time.

Platelet volume over time :

Platelet volume did not significantly evolve over time. The statistical difference between KI-, WT- or HET-mice (see Fig. 2B) remained steady at all time-points. Between HET- and WT-mice, differences were statistically significant at all time-points except the 4 month time-point.

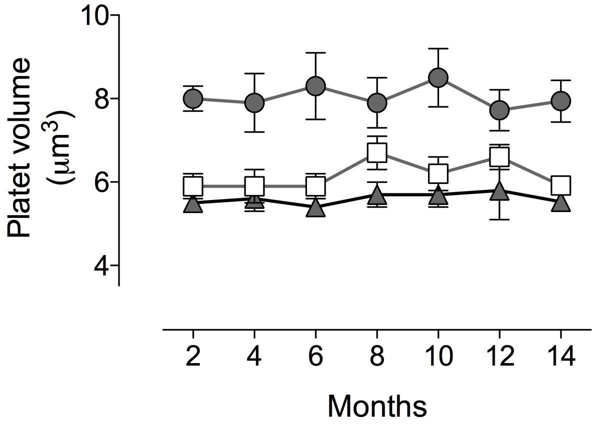

Figure S3 : Follow-up of platelet volume over time. Means ± SD measured in WT- (triangles), HET- (open squares) and KI-(circles) mice.

Perfusion of platelets over freshly released VWF

Perfusion of fixed platelets over VWF secreting endothelial cells resulted in the formation of platelet-decorated VWF-strings (Fig. S4). For VWF secreted from wild-type human umbilical vein endothelial cells (WT-HUVECs), the mean platelet coverage was 0.15±0.03 platelets/μm (mean±SD; n=6 experiments; Fig. S4A). Interestingly, platelet adhesion was markedly increased when platelets were perfused over HUVECs expressing VWF/p.R1306Q: 0.35±0.11 platelets/μm (n=6; p=0.0057; Unpaired two-tailed t test with Welch's correction; Fig. S4B).

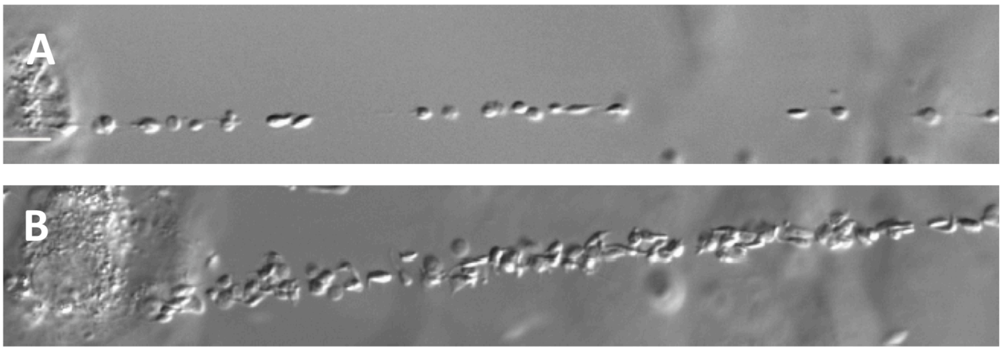

Figure S4 : Fixed human platelets were perfused over WT-HUVECs (panel A) and HUVECs obtained from a newborn homozygous for the VWD-type 2B mutation p.R1306Q (panel B). Bar represents 8 µm.

Supplementary methods belonging to supplementary figure S4: HUVECs from healthy newborns and from a homozygote newborn with VWD-type 2B (mutation p.R1306Q) were isolated as described[1] and cultured in endothelial basal medium (EBM)-2 supplemented with EGM-2 bulletkits (Lonza, Walkersville, MD, USA). For perfusion studies, HUVECs were grown on glass cover slips till >80% confluence. Prior to perfusion, coverslips were washed once in Medium 199 (Hyclone/ThermoFischer, Waltham, MA, USA). Fixed platelets (150.000/µL) were perfused over the cells at a shear rate of 300 s^{-1} as described[2]. Formation of VWF strings was visualized using an Axio Observer Microscope (Zeiss, Oberkochen, Germany). After 2.5 minutes of perfusion, a snapshot was taken for quantification of the amount of platelets that adhered to VWF. Platelet adhesion is expressed a number of platelets/µm of string. Each experiment was analyzed independently by two individuals.

Self-association of VWF-fragments to full-length VWF

When testing for binding of A1-Fc or A1-A2-A3-HPC4 to wt-VWF, we could not detect any interaction in our conditions. In contrast, a dose-dependent association of both VWF-

fragments to VWF/p.V1316M became apparent. This suggests that the type 2B mutation p.V1316M promotes inter-molecular VWF-VWF interactions.

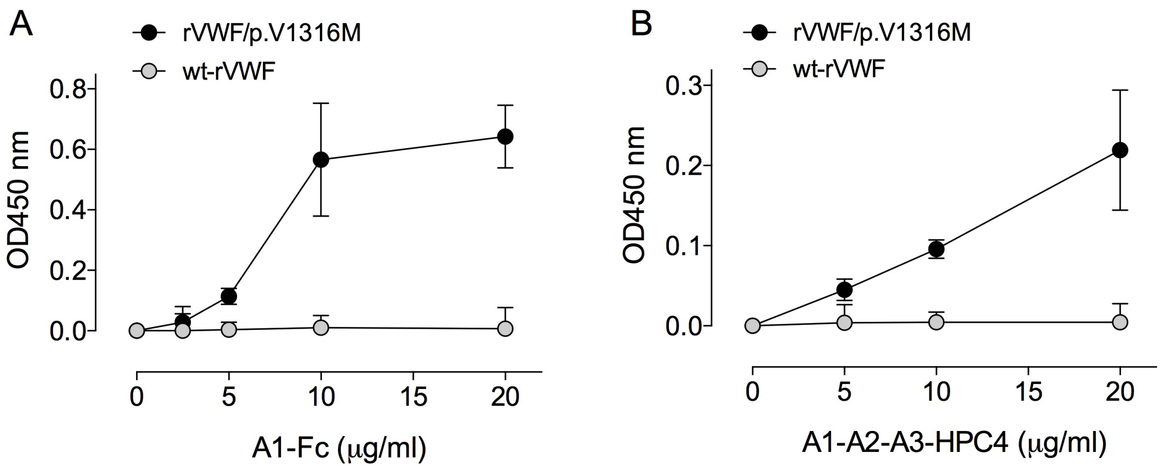

Figure S5: Self-association of VWF-fragments to full-length VWF. Dose-dependent association of the VWF A1-Fc fragment (panel A) or the VWF A1-A2-A3-HPC4 fragment (panel B) to the type 2B mutant VWF/p.V1316M. No relevant binding to wt-VWF could be detected under these conditions.

Supplementary methods belonging to supplementary figure S5: Culture medium containing full-length recombinant wt-VWF or VWF/p.V1316M (both 1 μg/mL final concentration) were incubated with either purified A1-Fc or A1-A2-A3-HPC4 (both 0-20 μg/mL) in Tris-buffered saline in eppendorf tubes. Binding is allowed to proceed for 15 minutes at room temperature on a MixMate® PCR mixer (Eppendorf, Montesson, France) at a rotation rate of 1,500 rpm. Samples were then transferred to microtiter-wells coated with the in-house made anti-D’D3 antibody Mab418 (5 μg/well) to catch VWF and VWF-fragment complexes. To prevent complexes from dissociating, the microtiter plate was also subjected to rotation on the MixMate® at 500 rpm. After washing to remove unbound protein, complexes were probed using peroxidase-labeled anti-human Fc or anti-HPC4 antibodies.

Determination murine ADAMTS13 activity

No significant difference in ADAMTS13 activity was measured between mice of the three genotypes.

Table S1 ADAMTS13 activity in WT-, HET- and KI-mice

	% ADAMTS13 activity	SEM	N
WT	109.4	2.6	8
HET	101.0	3.0	10
KI	108.7	4.8	10

Supplementary References :

1 Willems, C. *et al.* Media conditioned by cultured human vascular endothelial cells inhibit the growth of vascular smooth muscle cells. *Experimental cell research* **139**, 191-197 (1982).

2 Sixma, J. J., de Groot, P. G., van Zanten, H. & M, I. J. A new perfusion chamber to detect platelet adhesion using a small volume of blood. *Thrombosis research* **92**, S43-46 (1998).
